# Trinucleotide Base Pair Stacking Free Energy for Understanding TF-DNA Recognition and the Functions of SNPs

**DOI:** 10.3389/fchem.2018.00666

**Published:** 2019-01-18

**Authors:** Gen Li, Yuan Quan, Xiaocong Wang, Rong Liu, Lihua Bie, Jun Gao, Hong-Yu Zhang

**Affiliations:** Hubei Key Laboratory of Agricultural Bioinformatics, College of Informatics, Huazhong Agricultural University, Wuhan, China

**Keywords:** base stacking, free energy, single nucleotide polymorphisms, molecular dynamics simulation, binding specificity, transcription factor

## Abstract

Single nucleotide polymorphisms (SNPs) affect base pair stacking, which is the primary factor for maintaining the stability of DNA. However, the mechanism of how SNPs lead to phenotype variations is still unclear. In this work, we connected SNPs and base pair stacking by a 3-mer base pair stacking free energy matrix. The SNPs with large base pair stacking free energy differences led to phenotype variations. A molecular dynamics (MD) simulation was then applied. Our results showed that base pair stacking played an important role in the transcription factor (TF)-DNA interaction. Changes in DNA structure mainly originate from TF-DNA interactions, and with the increased base pair stacking free energy, the structure of DNA approaches its free type, although its binding affinity was increased by the SNP. In addition, quantitative models using base pair stacking features revealed that base pair stacking can be used to predict TF binding specificity. As such, our work combined knowledge from bioinformatics and structural biology and provided a new understanding of the relationship between SNPs and phenotype variations. The 3-mer base pair stacking free energy matrix is useful in high-throughput screening of SNPs and predicting TF-DNA binding affinity.

## Introduction

The stacking of adjacent base pairs and the pairing between complementary bases via hydrogen bonding are fundamentally related to the sequence and shape properties of DNA and critically influence the configurations, stabilities, and other properties of DNA(Yakovchuk et al., 2006; Hase and Zacharias, 2016; Kilchherr et al., 2016). The structure of a DNA sequence at the transcription factor (TF) binding site affects the interactions between these molecules (Rohs et al., 2009; Stormo and Zhao, 2010). In addition to current methodologies (Garvie and Wolberger, 2001; Slattery et al., 2014), base pair stacking free energy provides a novel way for understanding TF-DNA interactions. Determining base pair stacking free energy for a DNA sequence is critical for substantiating this underlying relationship. In the past few decades, several methods have been developed for determining base pair stacking free energy, including optical spectroscopic techniques (Warshaw and Tinoco, 1966), NMR spectroscopy (Chan and Nelson, 1969), and self-diffusion NMR (Stokkeland and Stilbs, 1985). More recently, base pair stacking free energies were measured in DNA fragments, such as beacon kinetics (Aalberts et al., 2003), thermal denaturation (Guckian et al., 1996), and mechanical unzipping (Huguet et al., 2010).

A single nucleotide polymorphism (SNP) is a common mutation phenomenon in the human genome (Clamp et al., 2007; Kimchi-sarfaty et al., 2007; Lu et al., 2015) and can significantly influence interactions between DNA and TFs, leading to related disease or phenotype variations (Deplancke et al., 2006; Bass et al., 2015). A single mutation in a SNP affects the base pair stacking free energies of two consecutive dinucleotides (2-mers), which compose a trinucleotide (3-mer) with the mutation site located at the central position. Studying 3-mers provides more comprehensive information than studying two 2-mers (Santalucia and Hicks, 2004; Taghavi et al., 2017). Another reason of selection 3-mer is because 3 base pairs are the minimal unit to describe the base-pairs stacking change of single SNP. However, measuring base pair stacking free energy for 3-mers is beyond the capability of current experimental approaches and remains a challenge to the scientific community (Sponer et al., 2013).

Base pair stacking is the primary factor for maintaining the stability of DNA structures (Yakovchuk et al., 2006). MD simulations (Hase and Zacharias, 2016) and quantum mechanics (QM) calculations (Parker et al., 2013) reveal that changes in the base pair stacking free energy affect many DNA parameters, including the twist, slide, groove, and bend of DNA. Recent studies showed that the structural changes caused by SNPs affect the binding affinity of protein-DNA complexes (Arkova et al., 2016). Based on the view that shape readout plays an important role in TF-DNA interactions (Slattery et al., 2011; Gordan et al., 2013; Yang et al., 2014), Zhou et al. developed a quantitative model that utilized sequences as well as DNA shape features and achieved a higher accuracy than traditional sequence models (Zhao et al., 2012; Mordelet et al., 2013; Zhou et al., 2015). Thus, we hypothesize that changes in the base pair stacking could cause DNA structural variations and transcriptional regulation disorders, which would eventually disrupt the TF-DNA interactions and lead to various diseases or phenotype variations. Therefore, measuring base pair stacking free energy is greatly beneficial for studying the underlying relationship between base-pair stacking and related disease or phenotype variations.

In this study, a 3-mer base pair stacking free energy matrix was constructed to calculate the base pair stacking free energy of 3-mers based on those of 2-mers. A 3-mer base pair stacking free energy difference matrix was then built to establish the correlation between the base pair stacking free energy and SNPs. Statistically significant variants from the GWAS database (GWASdb) were analyzed to identify the relationship between the base pair stacking free energies and the SNPs related to phenotype variations. The phenotype variations were enriched in the regions that possessed large differences in the base pair stacking free energies. Next, MD simulations revealed that changes in the base pair stacking free energies led to function variations via structural changes in the DNA, including twist, slide, and groove. Lastly, base pair stacking free energy was combined with experimental sequence data to generate a 1-mer+Δ*G*_*s*_ model for quantitatively predicting TF-DNA binding affinities, which exhibited a higher accuracy efficiency than 1-mer model. Our study revealed the significance of base pair stacking free energies for tri- or longer nucleotides and their relationships with the function of SNPs. We believe that the 3-mer base pair stacking free energy matrix may pave a new way for understanding and predicting TF-DNA interactions.

## Materials and Methods

### Generation of the 3-mer Base Pair Stacking Free Energy Matrix

A base pair stacking interaction involves adjacent base pairs, which means we need two 2-mers to represent a complete stacking interaction. Although 2-mer base pair stacking has been studied (Warshaw and Tinoco, 1966; Chan and Nelson, 1969; Stokkeland and Stilbs, 1985; Guckian et al., 1996; Aalberts et al., 2003; Huguet et al., 2010), 3-mer base pair stacking is poorly studied, partly due to the limitations of experimental techniques and the computing power of quantum mechanics (Sponer et al., 2013). To calculate the 3-mer base pair stacking free energy, we combined the base pair stacking free energies for the two consecutive 2-mers in the 3-mer (Santalucia and Hicks, 2004; Taghavi et al., 2017):

(1) $$\begin{array}{l} \Delta G_{ABC}=\Delta G_{AB}+\Delta G_{BC} \end{array}$$

where Δ*G*_*ABC*_ is the base pair stacking free energy of three consecutive nucleotides, ABC, and Δ*G*_*AB*_ and Δ*G*_*BC*_ are the stacking free energies of two adjacent nucleotides within the three consecutive nucleotides, AB and BC, respectively. Our 3-mer base pair stacking free energy matrix was constructed using the 2-mer base stacking energies from Protozanova et al.'s experimental data (Protozanova et al., 2004).

### Building the Phenotype Variation Related 3-mer SNPs Dataset

We downloaded SNPs related to human phenotypes (traits) from the GWASdb (http://jjwanglab.org/gwasdb, before August 2015). The GWASdb is the most widely used GWAS result database (Li et al., 2012), and it combines the National Human Genome Research Institute (NHGRI) GWAS Catalog, the tables and supplementary materials of manuscripts archived in the NHGRI GWAS Catalog, and the database of Genotypes and Phenotypes (dbGaP). To obtain the SNPs notably related to phenotype variations, only statistically significant (*P* ≤ 1 × 10^−8^) variants were included. A total of 25,029 SNPs, which included 883 human traits, were used for the analysis. To study the changes in the 3-mer base pair stacking free energy caused by the SNPs, the SNP adjacent nucleotides were obtained from the BioMart of Ensembl database (version 88) (Yates et al., 2016). To analyze the SNPs that were located at TF binding sites, we used RegulomeDB (http://www.regulomedb.org) to filter out the SNPs in the motif (Boyle et al., 2012), and there were 10123 SNPs in total. The raw data can be found in the Supplementary Data Sheet.

### Building the High-Resolution TF-DNA Complex Crystal Structure and JASPAR Dataset

The crystal structures of the TF-DNA interaction complexes used in this work were obtained from the Protein Data Bank and were published before 11 January 2017. There were 81 crystal structures that contained both TF and DNA with ≤ 2.0 Å resolution and no chemical modifications, mismatches or drugs. All of the TF-DNA interaction crystal structure complexes are listed in Table S1. The TFBS dataset contains nucleotide sequences that are within a distance of 3.5 Å of the TF in the high-resolution TF-DNA complex crystal structure dataset. The JASPAR database dataset consists of 593 non-redundant core nucleotide sequences in the JASPAR database that were download from http://jaspar.binf.ku.dk.

According to the work by Bass et al. (2015), we first selected the SNPs related to human phenotype variations with the highest supporting evidence score. Second, the corresponding TF-DNA complexes from our high-resolution TF-DNA complex crystal structure dataset were screened. Only 2 SNPs (MUT_17 and MUT_190) were reserved. MUT_17 was located in a specific binding site of Hepatocyte nuclear factor 4 alpha (HNF4α), a transcription factor containing zinc finger motifs, with force field parameters that still need improvements (Santos-martins et al., 2014). MUT_190 was located in a specific binding site of MEIS1. The structure of MEIS1 is similar to that of HNF4α, which contains a DNA binding domain but not a zinc finger (Jolma et al., 2015). There is an A → G SNP in the binding site (ACT*A*TCGA → ACT*G*TCGA) that is located in the MCP-1 promoter sequence (−2511 to −2528) at −2518, and this SNP increases MEIS1 binding affinity and leads to hepatitis C virus (HCV)-related liver disease (Bass et al., 2015). The MEIS1 complex (PDB ID: 4XRM) was adopted as a template to construct the structures of mutated-complexes.

### Molecular Dynamics Simulation Protocol

The MD simulations of the constructed systems were performed by using the NAMD software package (Kal et al., 1999) with AMBER ff14SB (Hornak et al., 2006) and parmbsc1 force fields (Ivani et al., 2016). Both the wild-type and mutated complexes and the free DNA were embedded in a box-shaped (72 × 68 × 84 Å^3^) bath of water molecules, and there was a layer of TIP3P water 12 Å in each direction from the atom with the largest coordinate in that direction. The system was neutralized with sodium cations. Na^+^ and Cl^−^ ion pairs were then added to reach a physiological salt concentration of 0.15 M. The solvated complex was equilibrated by carrying out a series of 1,000 steps of energy minimization with 10 kcal/mol/Å^2^ restraints on the backbone, after 1000 steps of minimization without restraints, 310 ps of heating restricted 2 kcal/mol/Å^2^ on the backbone from 0 to 310 K and 1 ns of density equilibration with NVT followed by 200 ns of constant pressure equilibration at 310 K. The system was equilibrated using an NPT ensemble at 310 K and pressure at 1 atm (1 atm = 101.3 kPa). All the simulations were run with SHAKE on hydrogen atoms, a 2 fs time step and a Langevin thermostat for temperature control and pressure control. Periodic boundary conditions and the Particle-Mesh-Ewald (PME)(Essmann et al., 1998) algorithm were adopted to compute the long range electrostatic forces, and the cutoff was set as 12 Å. Trajectory frames were collected at every 5 ps for a total of 50 ns. Curves+ software (Swaminathan et al., 1990; Blanchet et al., 2011) was employed to calculate the base pair parameters to define the geometry of the DNA. The values of the MD geometries presented here ignore the terminal base pairs of the oligomers since these often suffer from local deformations (Etheve et al., 2016). The standard value for the DNA structures used the data from Olson et al. (2001).

### Model of TF Binding Affinity Prediction

For a DNA sequence of length K, the 1-mer feature was used to represent each nucleotide position, and the target sequence was seen as a binary vector with a length of 4K. For example, one nucleotide position was encoded as 0 0 0 1, which indicated A, T, G, and C, respectively, and a value of 1 represented the occurrence. Regarding the base pair stacking free energy feature, a sliding-window approach to the DNA sequence calculated the base pair stacking with every 3-mer. We used the genomic context PBM (gcPBM) data from Zhou et al. to train and test the 1-mer model, the 1-mer+shape model, and the 1-mer+Δ*G*_*s*_ model. The gcPBM was derived from the Gene Expression Omnibus (GEO) under the accession number GSE59845 (Zhou et al., 2015), which contained 36-bp genomic sequences. The gcPBM data for each TF were converted into a matrix after preprocessing and feature encoding. The first column of this matrix contained the natural logarithm of the fluorescence signal intensities of the PBM probes, and the remaining columns contained the encoded features. The E-SVR algorithm in the LIBSVM toolkit (Chang and Lin, 2011; Claesen et al., 2014) was used to train the linear regression model to predict the natural logarithm of the gcPBM signal intensities based on the encoded sequence and base pair stacking free energy. The total length of the DNA base pair stacking vectors was 34 due to the unavailability of the values at two positions at the end. To obtain unbiased performance estimates of the regression models in each dataset, a nested 10-fold cross-validation procedure was implemented. The details of the gcPBM raw data processing methods were described by Zhou et al. (2015). The time-consuming of the model were tested on CPU E5-2683v3.

## Results and Discussion

### Building the 3-mer Base Pair Stacking Free Energy Matrix

The base pair stacking free energies for 3-mers were calculated by the sum of the base pair stacking free energies for two consecutive 2-mers (see Equation 1 in the Materials and Methods section). This strategy was used also by SantaLucia et al. (Santalucia and Hicks, 2004) and Taghavi et al. (2017) and showed reliable accuracies. Currently, base pair stacking free energies for 2-mers have been extensively studied, both theoretically and experimentally. Friedman and Honig calculated 2-mer base pair stacking free energies theoretically and reported values ranging from −7.79 to −4.36 kcal/mol (Friedman and Honig, 1995), whereas, Protozanova et al. measured them experimentally in a nicked DNA duplex, with values ranging from −2.17 to −0.19 kcal/mol (Protozanova et al., 2004), and later, Kilchherr et al.'s experimental results values ranged from −3.42 to −0.78 kcal/mol (Kilchherr et al., 2016). Since the theoretical 2-mer stacking free energies have a tendency for overestimations (Hase and Zacharias, 2016), we used the 2-mers stacking free energies from Protozanova et al.'s study to calculate the 3-mer base pair stacking free energies for their wide acceptances (Hase and Zacharias, 2016).

**Figure 1:**
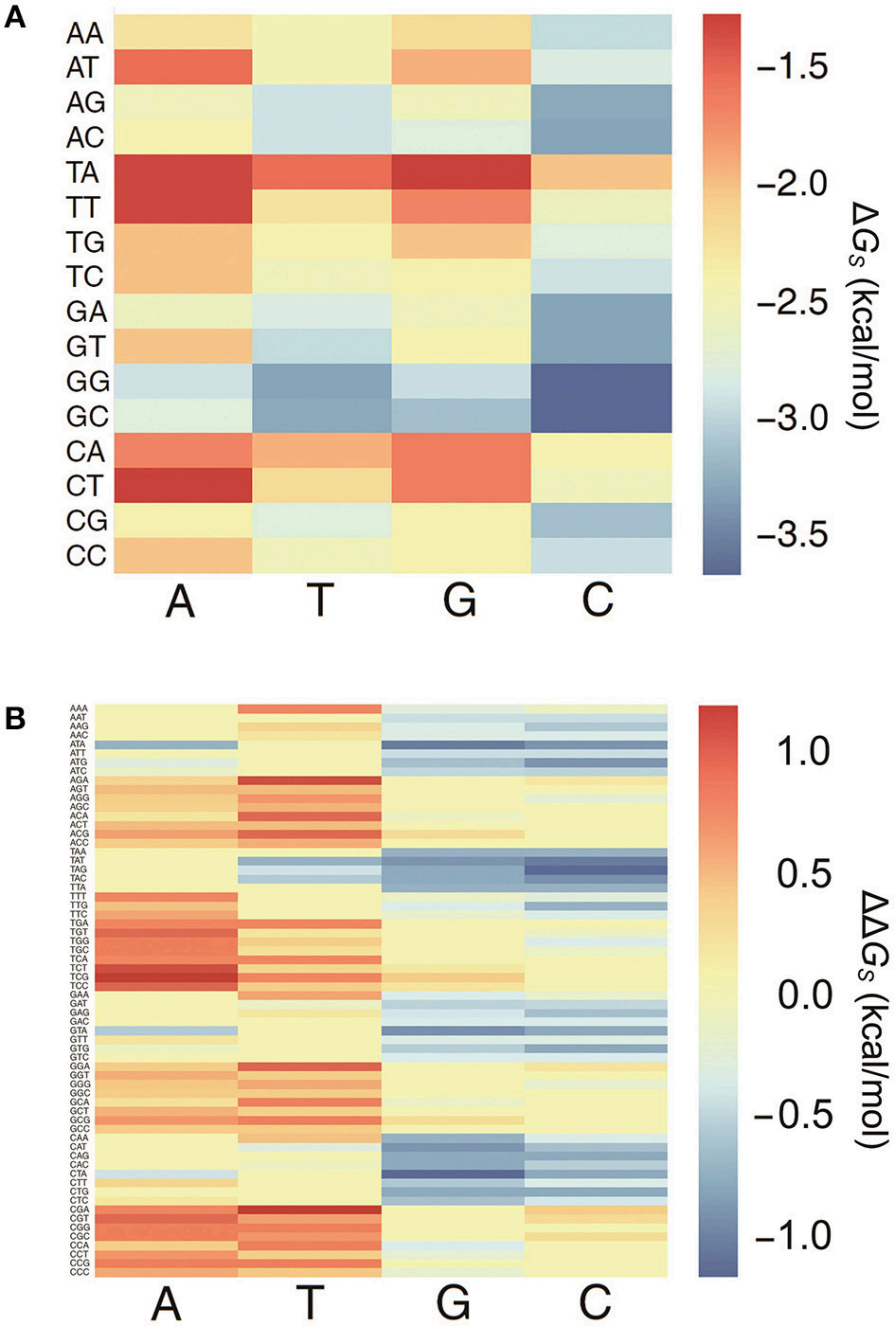
The heat map of 3-mer base pair stacking matrixes. **(A)** Heat map of the 3-mer base pair stacking free energy matrix. The X-axis is the last nucleotide in the 3-mer, and the Y-axis is the first two nucleotides. **(B)** Heat map of the 3-mer base pair stacking free energy difference matrix. The X-axis is the mutation at the middle position in the 3-mer, and the Y-axis is the 64 combinations of 3-mers. The values for the individual entries in the 3-mer base pair stacking free energy matrix and the 3-mer base pair stacking free energy difference matrix are listed in Tables S2, S3.

All 64 combinations of the 3-mer base pair stacking free energies were computed, and a 3-mer base pair stacking free energy matrix was created (Figure 1A). Generally, the stacking interaction between the GC base pair is stronger than that between the TA base pair (Geggier and Vologodskii, 2010). In 2-mers, GC and TA have the most negative and positive values for base pair stacking free energies, respectively. Similarly, in 3-mers, GGC and GCC base pairs have the most negative base pair stacking free energies, while TAG and CTA have the most positive base pair stacking free energies.

A 3-mer base pair stacking free energy difference matrix was also developed to study the changes in the base pair stacking free energies for SNPs (ΔΔ*G*_*s*_). In this matrix, the changes in the base pair stacking free energies for 64 trinucleotides and 4 possible mutations at each central nucleotide were calculated from the 3-mer base pair stacking free energy matrix. As shown in Figure 1B, the base pair stacking free energies for the 3-mers decreased after mutations to G and C occurred and increased after mutations to A and T. The maximum changes were CTA → CGA (−1.09 kcal/mol) and TCG → TAG (+1.09 kcal/mol). Furthermore, the value of the ΔΔ*G*_*s*_ was always lower for a SNP with a mutation to G or C compared to the same SNP with a mutation to A or T.

### Investigating the Relationship Between the 3-mer Base Pair Stacking Free Energy Difference Matrix and the Phenotype Variation

Recent advances in genome-wide association studies (GWAS) in genetics have enabled us to identify thousands of genetic variants that are associated with phenotype variations. It is well known that SNPs are closely linked with various phenotypes or traits (Kimchi-sarfaty et al., 2007; Helyar et al., 2011; Gutierrez-arzaluz et al., 2017), such as obesity and age-related macular degeneration(Sangiovanni et al., 2017; Dong et al., 2018). Herein, several assumptions were made to explore the relationships between SNPs and phenotype variations.

The first assumption was that the enhanced of the base pair stacking free energy was related to the phenotype variation. To validate this assumption, a phenotype variation-related 3-mer SNPs dataset was generated. There were 25,029 SNPs in the dataset, which involved 883 human traits. The base pair stacking free energy difference of each 3-mer SNP was obtained via the 3-mer base pair stacking free energy difference matrix. Interestingly, 47.83% of the variants (Table S4) showed enhanced base pair stacking interactions after mutation, whereas this ratio increased to 51.87% (hypergeometric test, *P* < 10^−20^) when only including variants with mutations in the TF binding sites. This increase may imply that the TFs are more sensitive to enhanced of base pair stacking. To confirm this hypothesis, we constructed two datasets (Table 1). The first was a high-resolution crystal structure dataset of TF binding, which had 81 binding sequences from the TF-DNA crystal structure complexes. The second one was the DNA binding motifs from the JASPAR database, which had 593 sequences of the TF binding motif. The AT contents in these two datasets were 54.90 and 53.94%, respectively. Therefore, we concluded that the relatively high ratio of the TF binding site was from not only the enhanced of the base pair stacking but also the high AT content of the TF binding site since the AT pairs have more room for enhancing the base pair stacking interactions during mutations.

**Table 1:** The ratio of AT and GC in the two different datasets.

**Dataset**	**Ratio (%)**	
	**AT**	**GC**
TFBS	54.90(538)	45.10(442)
JASPAR database	53.94(772)	46.06(654)

*The TFBS dataset is the nucleotide sequences that were within a distance of 3.5 Å of the TF in our high-resolution TF-DNA complex crystal structure dataset. The JASPAR database dataset consists of the nonredundant core nucleotide sequences in the JASPAR database*.

**Figure 2:**
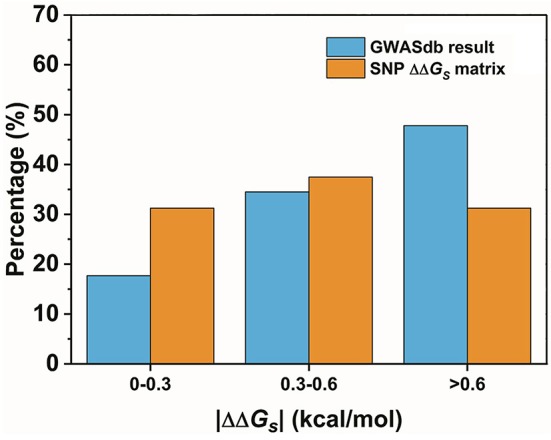
The distribution of the base pair stacking free energy differences. The SNPs of the TF binding sites are labeled in blue, and the distribution of the 3-mer base pair stacking free energy difference matrix is labeled in orange. Student's test of the two series yielded a value of *P* < 10^−9^.

**Figure 3:**
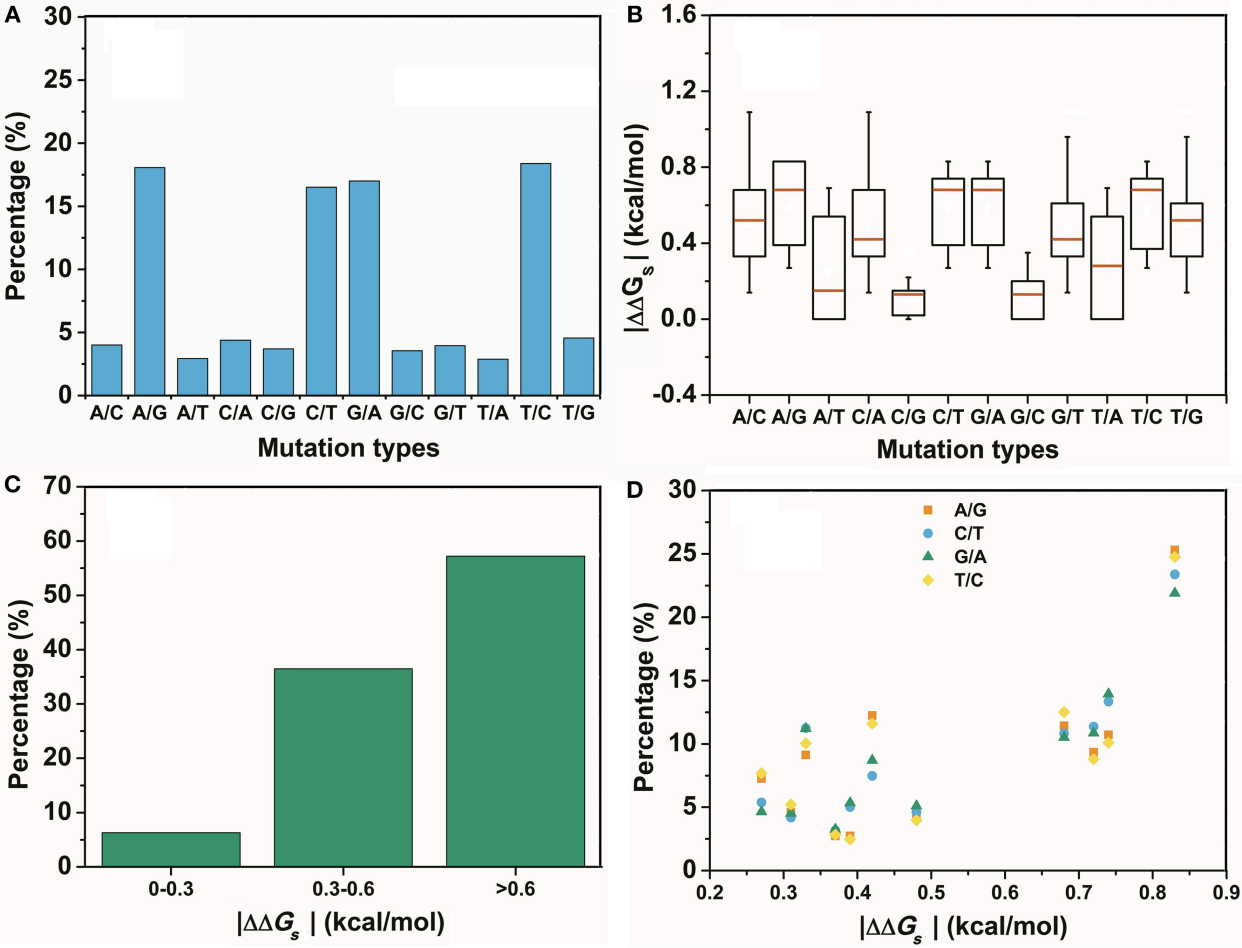
The distribution of the different mutation types. **(A)** The percentage of the different mutation types in the GWASdb result. **(B)** The average |ΔΔ*G*_*s*_| for the variants chosen from the GWASdb in the different mutation types. **(C)** The percentages of the variants in the top 4 mutation types of **(A)** with different |ΔΔ*G*_*s*_| ranges. **(D)** The distributions of the |ΔΔ*G*_*s*_| for the top 4 mutations types in **(A)**.

The second assumption was that the scale of the base stacking difference was related to the phenotype variation. First, we focused on the SNPs in TF binding sites, since it may affect TF-DNA interactions. The changes in the base pair stacking free energy (|ΔΔ*G*_*s*_|) of the phenotype variation-related 3-mer SNPs dataset (for TF binding site only) were obtained from the 3-mer base pair stacking free energy difference matrix and were manually screened. As shown in Figure 2, we categorized the variants of the |ΔΔ*G*_*s*_| values into three bins, namely, 0.0–0.3, 0.3–0.6, and larger than 0.6 kcal/mol. The ratios of the |ΔΔ*G*_*s*_| values were 17.7, 34.5, and 47.8%, respectively. Interestingly, the total number of the SNPs with a |ΔΔ*G*_*s*_| value lager than 0.6 kcal/mol was almost half of all the phenotype variation-related SNPs (the overall SNP results that had the same trend as the SNP located at the TF binding sites are found in Figure S1). At the same time, we calculated the |ΔΔ*G*_*s*_| value distribution of the 3-mer base pair stacking free energy difference matrix. It was nearly evenly distributed, and the ratio of >0.6 kcal/mol was ~30%. Student's test on the two series yielded a value of *P* < 10^−9^, indicating that the SNPs located in TF binding sites with a larger |ΔΔ*G*_*s*_| had a higher ratio. To confirm this finding, the SNPs were recategorized based on their mutation types (Figure 3A). There was a marked preference A → G, C → T, G → A, and T → C mutations, which accounted for 70.0% of all SNPs. Interestingly, the average |ΔΔ*G*_*s*_| values for these four types of mutations were also the top 4, as shown in Figure 3B. Furthermore, as shown in both Figures 3C,D, the distribution of the |ΔΔ*G*_*s*_| for SNPs in the 4 preferred mutation types displays a similar trend to that found in all the chosen variants, in which a larger |ΔΔ*G*_*s*_| occupies a higher ratio.

**Figure 4:**
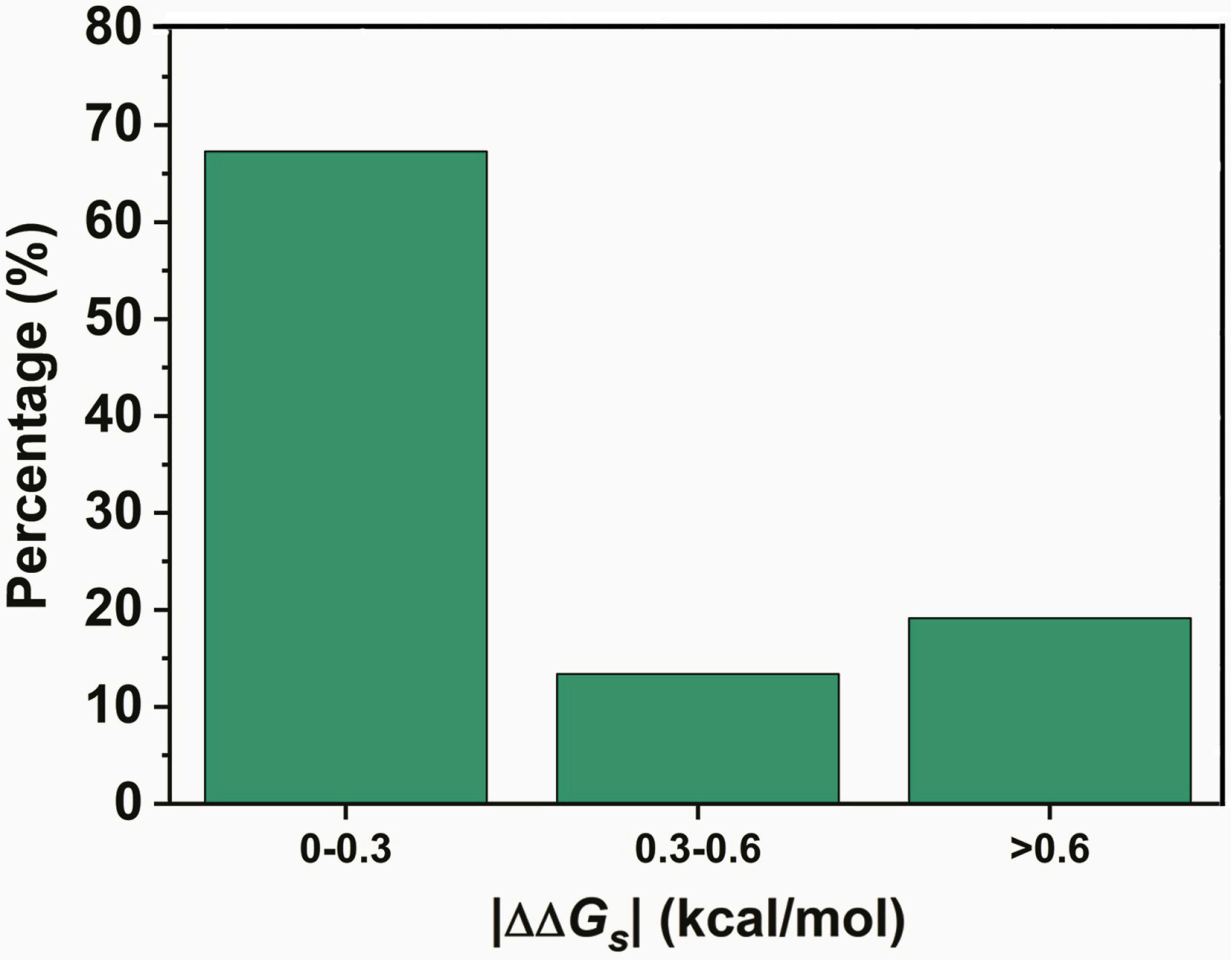
The percentages of the variants in the 2 mutation types with the lowest ratio mutation types with different |ΔΔ*G*_*s*_| ranges.

In contrast, SNPs with a low |ΔΔ*G*_*s*_| were speculated to be disfavored. A total of 590 variants in the two lowest ratio mutation types displayed a significantly different distribution from those in the preferred mutation types. Variants with a |ΔΔ*G*_*s*_| smaller than 0.3 kcal/mol accounted for over 70% of the total in these two mutation types (Figure 4). Therefore, SNPs with a smaller |ΔΔ*G*_*s*_| appeared to be disfavored in our GWASdb results. Since all of our chosen variants were statistically significant, this implied that the SNPs in the TF-binding sites with a larger |ΔΔ*G*_*s*_| were more likely to lead to a phenotype variation. In summary, our study with statistically significant variants from the GWASdb showed that phenotype variation prefers SNPs with a large |ΔΔ*G*_*s*_| and made a solid case, where a 3-mer base pair stacking free energy matrix and a 3-mer base pair stacking free energy difference matrix helped to probe the relationship between base pair stacking free energy and diseases.

### Simulation of the Relationship Between Base Pair Stacking and TF-DNA Interactions

As discussed earlier, SNPs result in changes in base pair stacking free energies. Recent studies showed that the base pair stacking free energy is closely related to DNA structure (Luscombe et al., 2001; Baker and Grant, 2007; Gu et al., 2015), which means it is likely that base pair stacking free energies affect TF-DNA interactions via DNA structural changes. However, the impact of SNPs on the structure of DNA is poorly studied, especially the relationship between the change in the base pair stacking free energy caused by the SNP and TF-DNA interaction. To explore this potential relationship, we searched the entire PDB, but did not find a TF-DNA cocrystal complex that had crystal structures for both wild-type and mutated complexes. To solve this problem, we combined our existing high-resolution TF-DNA complex crystal structure dataset with the study by Bass et al. (2015). The SNPs with the highest supporting evidence scores listed in the study by Bass et al. were selected. These SNPs are all located in the regulatory region and have been studied for their influence on TF-DNA binding affinities (Bass et al., 2015). Only two TF-DNA complexes with these SNPs in the regulatory region have available cocrystal structures, namely, HNF4α and Meis homeobox 1 (MEIS1). However, HNF4α was excluded due to inaccurate force field parameters for the zinc finger motifs (Santos-martins et al., 2014).

**Figure 5:**
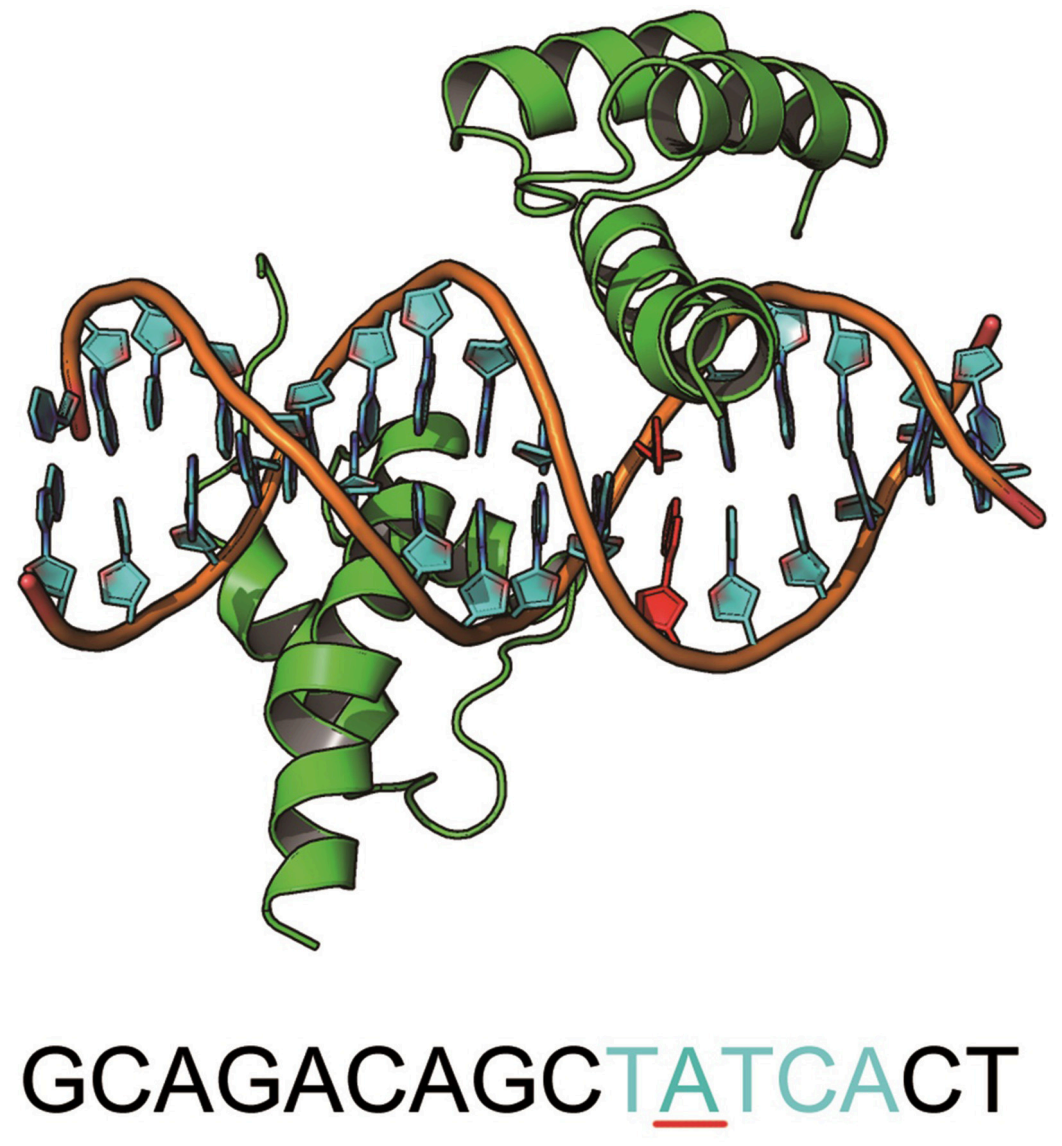
The structure of MEIS1 and its DNA. The blue DNA sequence is the motif, and the red nucleotide is the mutation site.

MEIS1 plays an essential role in the development and function of vertebrate organs (Shen et al., 1999). It is a homodimer of the TALE type homeobox transcription factor that regulates gene expression by binding specific DNA sequences (Jolma et al., 2015) (Figure 5). There is a SNP at the binding site (ACT**A**TCGA → ACT**G**TCGA) that causes an increase in the binding affinity and results in hepatitis C virus (HCV)-related liver disease (Bass et al., 2015). More interestingly, this mutation (A → G) was one of the 4 preferred mutation types in our chosen variants from the GWASdb (Figure 3A). The cocrystal structure for the MEIS1 complex (PDB ID: 4XRM) was adopted as a template to construct the structures of the mutated complexes.

To verify our molecular models for the MEIS1 complexes, both the Helmholtz (Δ*H*_*b*_) and binding free energies (Δ*G*_*b*_) for the wild-type and mutated complexes were calculated. As shown in Table S5, the Δ*H*_*b*_ for the mutated complex was −6.27 kcal/mol. After considering the entropic penalty (Chang et al., 2007), the Δ*G*_*b*_ for the mutated complex was still −1.42 kcal/mol. This was consistent with the experimental measurements, in which the binding affinity for the MEIS1 complex increases after the A → G mutation in the TF binding site (Bass et al., 2015). Our MM/GBSA results correlated well with the Montclare's DNA mutation binding affinity experiments, which report values ranging from −5.0 to −1.3 kcal/mol (Montclare et al., 2001).

**Figure 6:**
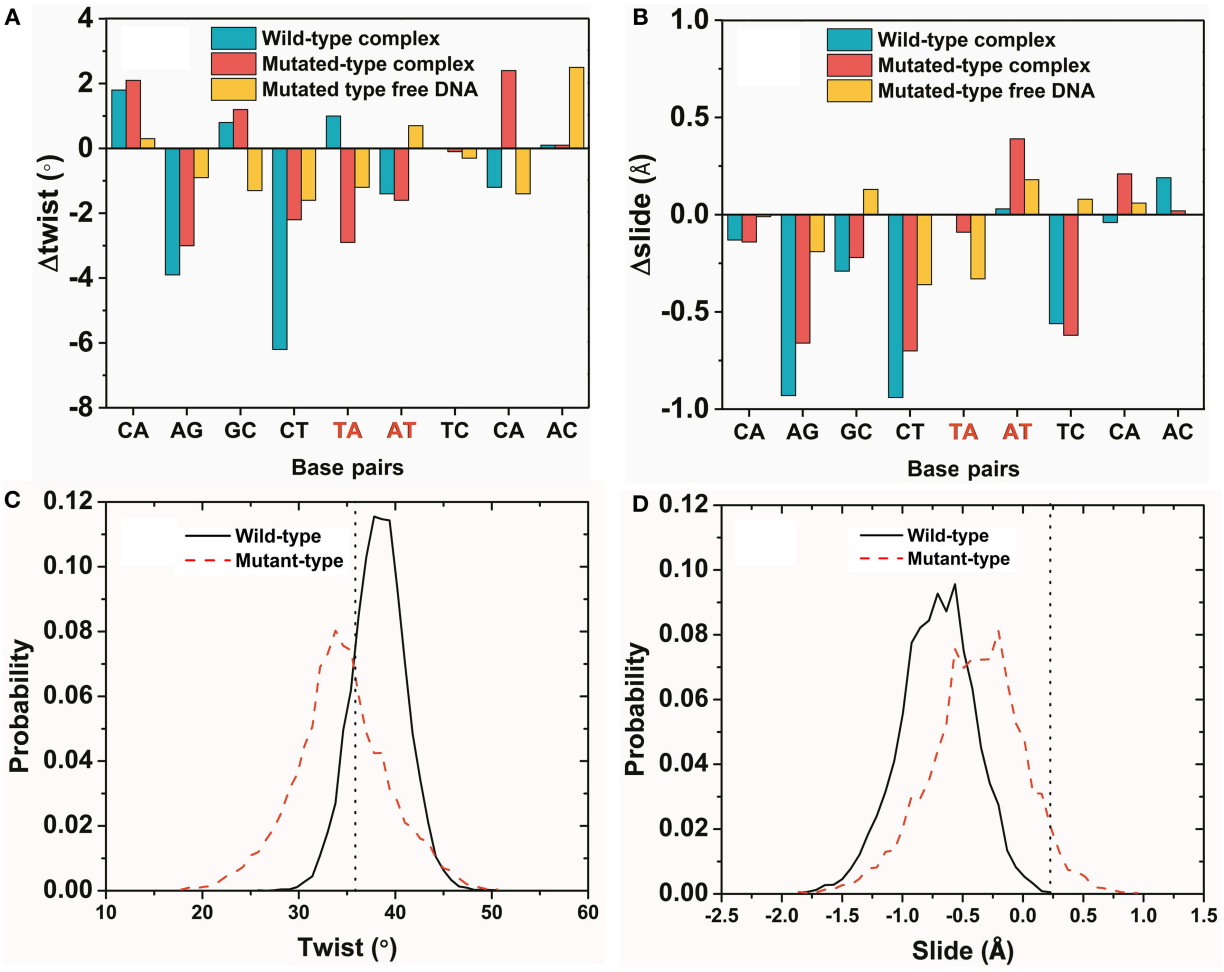
The variation of DNA structure parameters. **(A,B)** The difference (D-values) in the twist and slide for the wild-type and mutated complexes and the mutated free DNA were obtained from the average structures of last 50 ns of the simulations. The mutated site is marked in red. **(C,D)** The probability distribution of a twist and slide at the mutation site for the wild-type and mutated complexes. The vertical dotted line is the standard value of the twist and slide. Most of the DNA structures had the same phenomenon as the twist and slide (Figures S2, S3).

The average parameters of the DNA structures of the wild-type and mutated complexes were then compared (Blanchet et al., 2011). We analyzed the average structure difference values (D-values) of the DNA parameter between the wild-type complex, the mutated complex, the mutated free DNA and the wild-type free DNA. First, the differences (D-values) in the twist and slide for the wild-type and mutated complexes and the mutated free DNA at the mutation site were small, whereas those for other sites were relatively larger (Figures 6A,B). This phenomenon showed that the stronger base pair stacking free energy (Table 2) made the DNA structure closer to that of the free DNA, since the inter parameter was directly related to the base pair stacking free energy, and it also showed that the base pair stacking free energy was a long-range allosteric effect (Gu et al., 2015). Second, the amplitude of the variation for left and right at the mutation site was inconsistent. As shown, the changes in the left side were obviously larger than those in the right side. For the complexes, the D-values of the twist and slide had a larger amplitude of variation than the free DNA. This suggested that the asymmetry of the left and right was due to the TF interaction (Figure 5 shows that the left side of the mutation site was the binding location of the other monomer). Lastly, for the slide, there was a small difference between the wild-type and mutated complexes. However, the difference in the wild-type and mutated for the twist were more notable than those for the slide, which meant the effect of the SNP on the twist was greater than that on the slide for the TF-DNA interaction (Czapla et al., 2006; Cooper et al., 2008; Carvalho et al., 2014; Machado et al., 2015; Ngo et al., 2016). In addition, the average values of the probability distribution curves for both twist and slide for the mutated complex (Figures 6C,D) were closer to the standard values than those for the wild-type complex. In the meantime, the base pair stacking free energy calculated from the 3-mer base pair stacking free energy matrix for TGT in the mutated complex was more negative than that for TAT in the wild-type (Table 2). The more significant structural changes in the mutated MEIS1 complex than in the wild-type complex demonstrated a solid example that changes in the base pair stacking free energies resulted in DNA structural variation and altered the binding affinity for TF-DNA complexes. It also indicated that the interplay between protein and DNA plays an important role in the regulation of the variation of base stacking (Koshland, 1958; Ramakers et al., 2017).

**Table 2:** Twist, slide, and base pair stacking free energy for the wild-type and mutated MEIS1 complexes.

**Type**	**Twist^a^ (°)**	**Slide****^a^** **(Å)**	**Δ*G_***s***_*****^b^** **(kcal/mol)**
Wild-type	38.3	−0.73	−1.53 (TAT)
Mutated-type	34.4	−0.53	−2.36 (TGT)
Standard Value^c^	36.0	0.23	/

a *Twist and slide of the DNA structures obtained from the average structures*.

b *ΔG_s_ was the calculated by the 3-mer base pair stacking free energy matrix for the 3-mer at the mutation site*.

c The standard value for the DNA structures used the data from Olson et al., 2001

### Quantitative Modeling of TF Binding Specificities Using Base Pair Stacking

Protein-DNA binding is an essential biological process that is involved in DNA replication, restriction, and modification, transcriptional regulation, etc. (Halford and Marko, 2004). Increasing efforts have been made to understand how proteins recognize specific binding sites in the genome. Rohs et al. divided protein-DNA interactions into two major categories, including base readout and shape readout (Rohs et al., 2010). Base readout refers to proteins that recognize DNA by forming specific hydrogen bonds and hydrophobic contacts with bases in the major or minor grooves (Seeman et al., 1976), and shape readout refers to proteins that recognize DNA by sequence-dependent DNA structures and deformability (Travers, 1989; Shakked et al., 1994; Koudelka et al., 2006). Although high-throughput experimental methods, such as protein-binding microarrays (Berger et al., 2006), measure the binding affinities for tens of thousands of DNA sequences *in vitro* at the same time, it still takes extensive efforts to carry out these experiments. To achieve a higher efficiency, several theoretical models for predicting the TF-DNA binding were developed based on massive experimental data (Foat et al., 2006; Zhao and Stormo, 2011; Weirauch et al., 2013; Orenstein and Shamir, 2014). A 1-mer model, one of the earlier models, predicts TF-DNA binding affinity solely based on sequence information. This model displays a high efficiency, yet a low accuracy (R^2^ < 0.8) when compared to more sophisticated models (Mordelet et al., 2013). Recently, the 1-mer+shape model (Zhou et al., 2015) was developed, which combines the sequence with the DNA structural information, such as the minor groove widths, propeller twists, rolls, and helix twists, as input features to achieve a higher accuracy (R^2^ > 0.9) at the cost of a significantly increased computing time. The 1-mer+shape model requires not only predictions of the structural information, which are usually obtained from resource-hogging all-atom Monte Carlo simulations (Zhou et al., 2013), but also a significant amount of time to be trained and tested.

**Figure 7:**
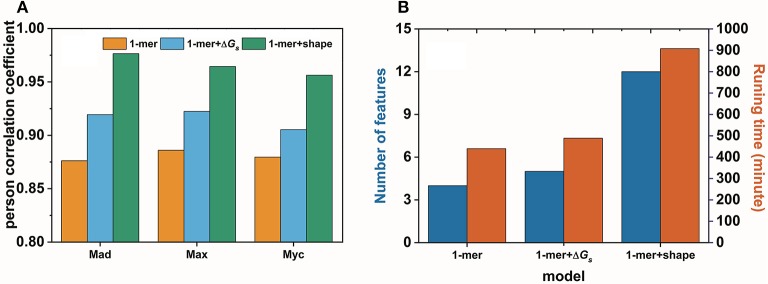
Performance **(A)** and efficiency **(B)** comparisons for the 1-mer, 1-mer+Δ*G*_*s*_, and 1-mer+shape models in predicting the binding affinities of Mad1 (Mxd1)–Max (Mad), Max–Max (Max), and c-Myc–Max (Myc) bound with DNA.

In this work, we confirmed, with an example, that base pair stacking free energy has an unambiguous relationship with TF-DNA interactions. In this respect, base pair stacking free energy might be used as a feature to predict TF-DNA binding affinity. Here, we extended the input matrix in the 1-mer model to include the base pair stacking free energies for all 3-mers in the DNA sequence to build the 1-mer+Δ*G*_*s*_ model. A 10-fold cross-validation was performed on the gcPBM data for three human basic helix–loop–helix TFs, including Mad1 (Mxd1)–Max (Mad), Max–Max (Max), and c-Myc–Max (Myc)(Mordelet et al., 2013), to compare our 1-mer+Δ*G*_*s*_ model with both the 1-mer and 1-mer+shape models. The person correlation coefficient (PCC) obtained by the 1-mer+Δ*G*_*s*_ model improved markedly compared with that of the 1-mer model. The PCC values were > 0.9 for all three TFs (Mad: PCC = 0.92, Max: PCC = 0.92, Myc: PCC = 0.91). Although the 1-mer+shape model demonstrated a higher Pearson correlation coefficient, it required significantly more features and running time (Figure 7A). By contrast, both the number of features per nucleotide position and the running time for the 1-mer+Δ*G*_*s*_ model were close to those required for the traditional 1-mer model (Figure 7B). Therefore, the 1-mer+Δ*G*_*s*_ model proved to be both accurate and efficient for predicting TF-DNA binding affinities, which might be exceedingly beneficial for preliminary disease screening.

Furthermore, as seen in the 3-mer base pair stacking free energy matrix, base pair stacking is sequence-dependent and is related to the DNA structures in the TF binding site. Thus, our 1-mer+Δ*G*_*s*_ model is deeply associated with the DNA's sequence and structural information, and the base pair stacking free energy, as a new prospect for understanding TF-DNA binding, has intrinsic connections to both base and shape readouts.

## Conclusions

Base pair stacking free energy is an essential property of DNA, and it is intrinsically associated with DNA sequences and shapes. Since the sequence and shape information have already been successfully employed to understand TF-DNA interactions, base and shape readouts, base pair stacking free energy provides a new prospect in this area. In the present study, we presented an unambiguous relationship between base pair stacking free energy and TF-DNA interactions. Both a 3-mer base pair stacking free energy matrix and a 3-mer base pair stacking free energy difference matrix were constructed for establishing the matrices between the SNPs and their base pair stacking free energies.

Our analyses of the variants from the GWASdb showed that mutations in SNPs with a larger |ΔΔ*G*_*s*_| had a higher probability of leading to a phenotype variation. MD simulations for the MEIS1 complexes demonstrated that the mutation in the TF-DNA binding site caused DNA structural changes and resulted in higher binding affinities. This mutation in the regulatory region was one of the four mutations with the largest |ΔΔ*G*_*s*_|, which suggested that changes in the base pair stacking free energy might lead to phenotype variations via DNA structural changes. Lastly, we generated the 1-mer+Δ*G*_*s*_ model to apply base pair stacking free energy for predicting TF-DNA binding affinities, and it exhibited a higher accuracy than the traditional 1-mer model and a high efficiency compared to the 1-mer+shape model.

Our molecular dynamics simulation also indicated that the interplay between protein and DNA is important to the regulation of base stacking. This was in consistent with our finding that the SNPs with a larger base pair stacking free energy change led to phenotype variations. This is because a larger base pair stacking free energy change might affect the protein-DNA interactions more easily. We believe that the 3-mer base pair stacking free energy matrix and the 3-mer base pair stacking free energy difference matrix are useful for high-throughput SNP screening and for predicting TF-DNA binding affinities. Furthermore, as demonstrated in this work, proteins also play an important role in the TF-DNA interaction, and how they effect of TFs is still an open question. Improving the precision of 3-mer base pair stacking free energy is now being carried out using the QM/MM method (Warshel and Levitt, 1976).

## Author Contributions

GL and JG write the manuscript. XW helped and partially write the manuscript. RL and LB partially did simulation of TF binding specificities. YQ and H-YZ contributed on analysis the functions of SNPs.

### Conflict of interest Statement

The authors declare that the research was conducted in the absence of any commercial or financial relationships that could be construed as a potential conflict of interest.
