# Supplementary material for: Trinucleotide Base Pair Stacking Free Energy for Understanding TF-DNA Recognition and the Functions of SNPs
Source: Front Chem. 2019 Jan 18;6:666. doi: 10.3389/fchem.2018.00666 (PMC6345724; doi:10.3389/fchem.2018.00666)
Supplement: Supplementary file 1 [file Table_1.DOCX]

Supplementary Material

# Supplementary Figures and Tables

## Supplementary Figures

**
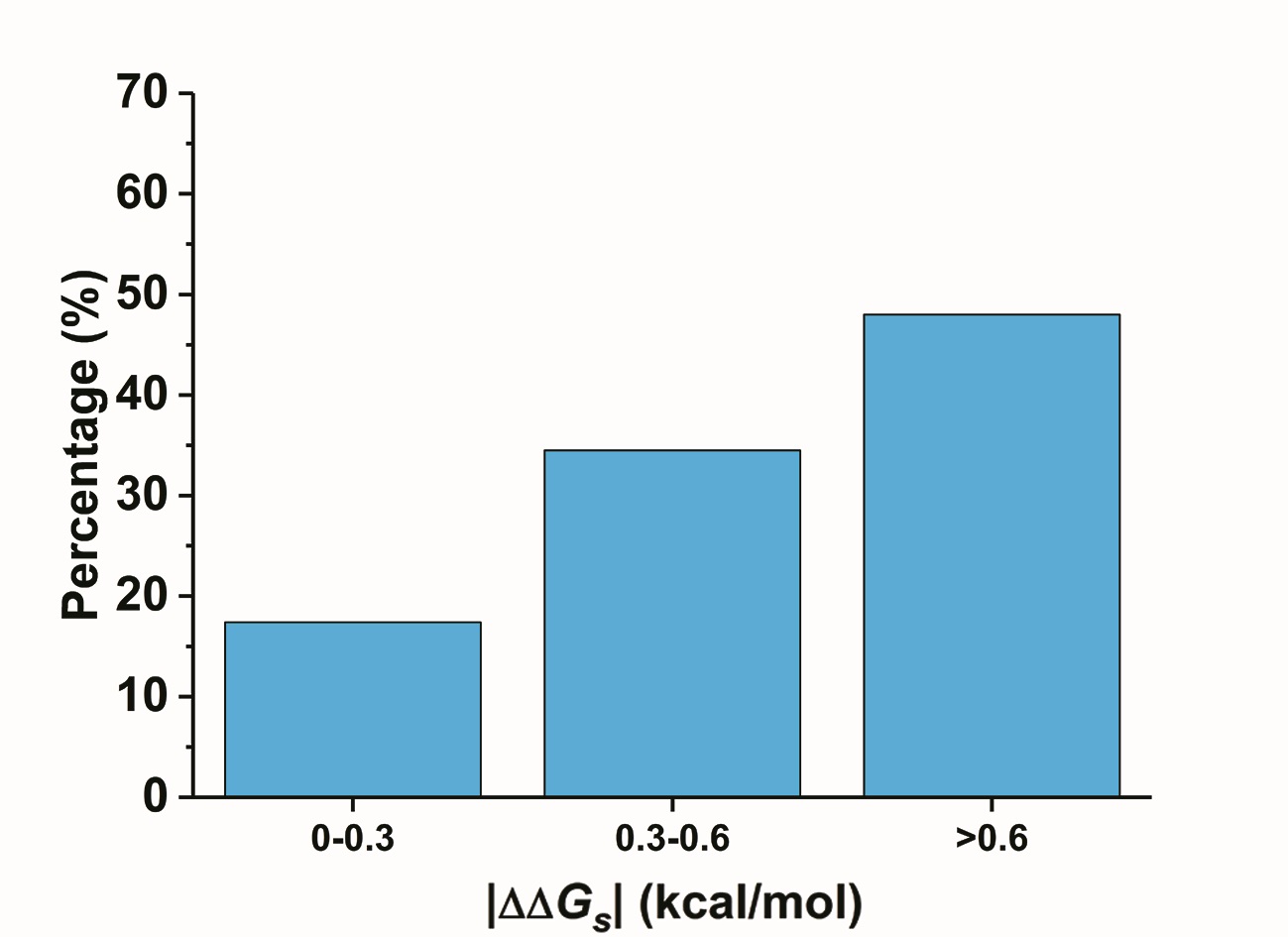
**

Figure S1 The distribution of the base stacking energy differences. SNPs are significantly related to the phenotype variations in the GWASdb.


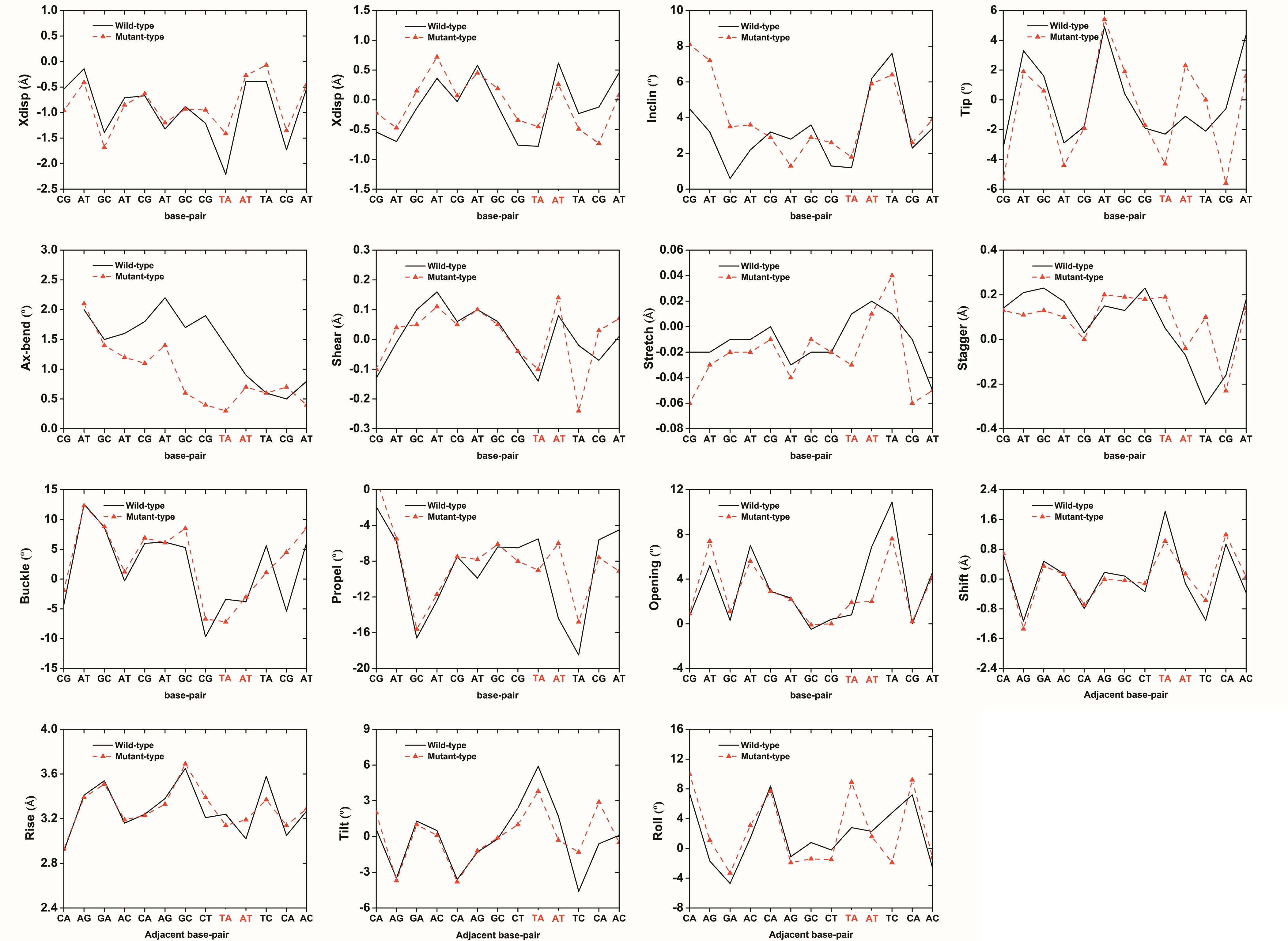


Figure S2 DNA structures of the wild-type (black solid curve) and mutated complexes (red dash curve) obtained from the average structure of the 50 ns equilibrium simulation.


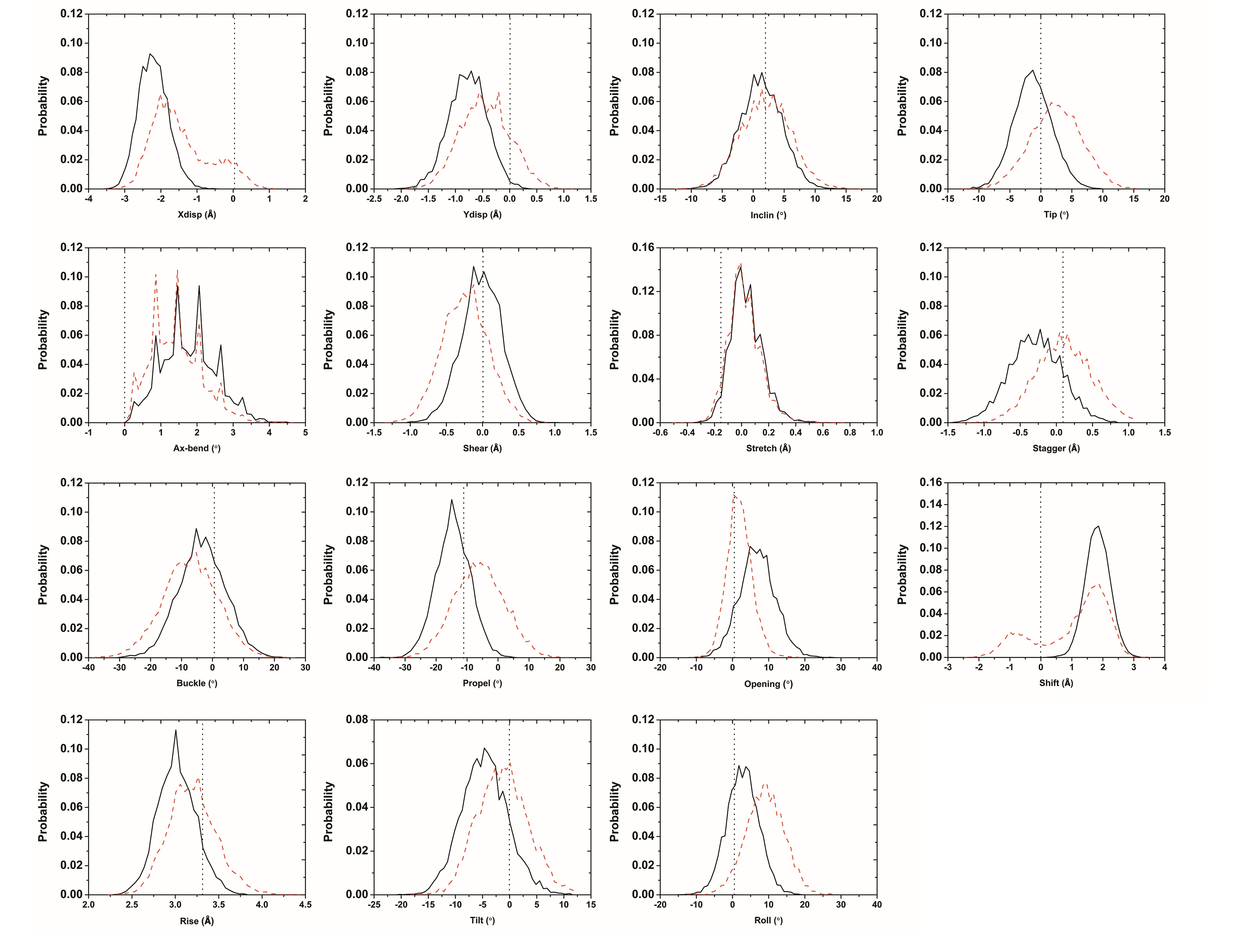


Figure S3 Probability distribution of twist and slide at the mutation site for the wild-type and mutated complexes. The vertical dotted line is the standard value.

Table S1 count summary of the TFs from the familial classification of the high-resolution TF-DNA complex crystal structure. This table lists the TF entries in the different families, whose structures, in complex with DNA and with a resolution higher than 2.0 Å, were available in the Protein Data Bank as of 11 January 2017. Some of the unreasonable structures were removed, such as a single chain or modified DNA. There were 81 crystal structures in total.

| TF family | Numbers | TF family | Numbers | TF family | Numbers |
| --- | --- | --- | --- | --- | --- |
| ARAR | 3 | CUT | 3 | FORK HEAD | 1 |
| BHLH | 3 | ZF-C2H2 | 16 | POU | 1 |
| BZIP | 1 | PAX | 1 | SLYA | 1 |
| CRP | 1 | IG-FOLD | 1 | SOX | 1 |
| C/EBP-RELATED | 2 | LAMBDA PHAGE CII PROTEIN | 1 | STEROID HORMONE RECEPTOR | 1 |
| ETS | 3 | LUXR | 1 | SU | 1 |
| FOX | 1 | MEF2 | 1 | TBP | 4 |
| GNTR/HUTC | 1 | MYB | 1 | TFIIB-LIKE | 1 |
| HD | 8 | NR | 16 | TRPR | 1 |
| CEBPB | 1 | P53 | 2 | ZF-GATA | 2 |

Table S2. Values of the 3-mer base pair stacking free energy matrix

| 1rd and the 2rd nucleotides | 3rd nucleotide | | | |
| --- | --- | --- | --- | --- |
|  | A | T | G | C |
| AA | -2.22 | -2.45 | -2.17 | -2.92 |
| AT | -1.53 | -2.45 | -1.89 | -2.77 |
| AG | -2.49 | -2.87 | -2.50 | -3.23 |
| AC | -2.36 | -2.87 | -2.72 | -3.25 |
| TA | -1.30 | -1.53 | -1.25 | -2.00 |
| TT | -1.30 | -2.22 | -1.66 | -2.54 |
| TG | -1.98 | -2.36 | -1.99 | -2.72 |
| TC | -1.98 | -2.49 | -2.34 | -2.87 |
| GA | -2.54 | -2.77 | -2.49 | -3.24 |
| GT | -2.00 | -2.92 | -2.36 | -3.24 |
| GG | -2.87 | -3.25 | -2.88 | -3.61 |
| GC | -2.72 | -3.23 | -3.08 | -3.61 |
| CA | -1.66 | -1.89 | -1.61 | -2.36 |
| CT | -1.25 | -2.17 | -1.61 | -2.49 |
| CG | -2.34 | -2.72 | -2.35 | -3.08 |
| CC | -1.99 | -2.50 | -2.35 | -2.88 |

The values of base pair stacking free energy in kcal/mol.

Table S3. Values of the 3-mer base pair stacking free energy difference matrix

| 3mer | mutation site was located at the center | | | |
| --- | --- | --- | --- | --- |
|  | A | T | G | C |
| AAA | 0.00 | -0.69 | 0.27 | 0.14 |
| AAT | 0.00 | 0.00 | 0.42 | 0.42 |
| AAG | 0.00 | -0.28 | 0.33 | 0.55 |
| AAC | 0.00 | -0.15 | 0.31 | 0.33 |
| ATA | 0.69 | 0.00 | 0.96 | 0.83 |
| ATT | 0.00 | 0.00 | 0.42 | 0.42 |
| ATG | 0.28 | 0.00 | 0.61 | 0.83 |
| ATC | 0.15 | 0.00 | 0.46 | 0.48 |
| AGA | -0.27 | -0.96 | 0.00 | -0.13 |
| AGT | -0.42 | -0.42 | 0.00 | 0.00 |
| AGG | -0.33 | -0.61 | 0.00 | 0.22 |
| AGC | -0.31 | -0.46 | 0.00 | 0.02 |
| ACA | -0.14 | -0.83 | 0.13 | 0.00 |
| ACT | -0.42 | -0.42 | 0.00 | 0.00 |
| ACG | -0.55 | -0.83 | -0.22 | 0.00 |
| ACC | -0.33 | -0.48 | -0.02 | 0.00 |
| TAA | 0.00 | 0.00 | 0.68 | 0.68 |
| TAT | 0.00 | 0.69 | 0.83 | 0.96 |
| TAG | 0.00 | 0.41 | 0.74 | 1.09 |
| TAC | 0.00 | 0.54 | 0.72 | 0.87 |
| TTA | 0.00 | 0.00 | 0.68 | 0.68 |
| TTT | -0.69 | 0.00 | 0.14 | 0.27 |
| TTG | -0.41 | 0.00 | 0.33 | 0.68 |
| TTC | -0.54 | 0.00 | 0.18 | 0.33 |
| TGA | -0.68 | -0.68 | 0.00 | 0.00 |
| TGT | -0.83 | -0.14 | 0.00 | 0.13 |
| TGG | -0.74 | -0.33 | 0.00 | 0.35 |
| TGC | -0.72 | -0.18 | 0.00 | 0.15 |
| TCA | -0.68 | -0.68 | 0.00 | 0.00 |
| TCT | -0.96 | -0.27 | -0.13 | 0.00 |
| TCG | -1.09 | -0.68 | -0.35 | 0.00 |

Table S3 (Continued )

| 3mer | mutation site was located at the center | | | |
| --- | --- | --- | --- | --- |
|  | A | T | G | C |
| TCC | -0.87 | -0.33 | -0.15 | 0.00 |
| GAA | 0.00 | -0.54 | 0.33 | 0.18 |
| GAT | 0.00 | 0.15 | 0.48 | 0.46 |
| GAG | 0.00 | -0.13 | 0.39 | 0.59 |
| GAC | 0.00 | 0.00 | 0.37 | 0.37 |
| GTA | 0.54 | 0.00 | 0.87 | 0.72 |
| GTT | -0.15 | 0.00 | 0.33 | 0.31 |
| GTG | 0.13 | 0.00 | 0.52 | 0.72 |
| GTC | 0.00 | 0.00 | 0.37 | 0.37 |
| GGA | -0.33 | -0.87 | 0.00 | -0.15 |
| GGT | -0.48 | -0.33 | 0.00 | -0.02 |
| GGG | -0.39 | -0.52 | 0.00 | 0.20 |
| GGC | -0.37 | -0.37 | 0.00 | 0.00 |
| GCA | -0.18 | -0.72 | 0.15 | 0.00 |
| GCT | -0.46 | -0.31 | 0.02 | 0.00 |
| GCG | -0.59 | -0.72 | -0.20 | 0.00 |
| GCC | -0.37 | -0.37 | 0.00 | 0.00 |
| CAA | 0.00 | -0.41 | 0.68 | 0.33 |
| CAT | 0.00 | 0.28 | 0.83 | 0.61 |
| CAG | 0.00 | 0.00 | 0.74 | 0.74 |
| CAC | 0.00 | 0.13 | 0.72 | 0.52 |
| CTA | 0.41 | 0.00 | 1.09 | 0.74 |
| CTT | -0.28 | 0.00 | 0.55 | 0.33 |
| CTG | 0.00 | 0.00 | 0.74 | 0.74 |
| CTC | -0.13 | 0.00 | 0.59 | 0.39 |
| CGA | -0.68 | -1.09 | 0.00 | -0.35 |
| CGT | -0.83 | -0.55 | 0.00 | -0.22 |
| CGG | -0.74 | -0.74 | 0.00 | 0.00 |
| CGC | -0.72 | -0.59 | 0.00 | -0.20 |
| CCA | -0.33 | -0.74 | 0.35 | 0.00 |
| CCT | -0.61 | -0.33 | 0.22 | 0.00 |
| CCG | -0.74 | -0.74 | 0.00 | 0.00 |
| CCC | -0.52 | -0.39 | 0.20 | 0.00 |

This table lists the ∆∆*G_s_* differences of the mutation site at the middle position in the 3-mer, and the values of ∆∆*G_s_* are in kcal/mol.

Table S4. The percentage of the phenotype variation caused by SNPs in the genome-wide and TF binding regions

| Mutated site region | ^a^positive ∆*∆G_s_* | ^b^negative ∆*∆G_s_* |
| --- | --- | --- |
| Genome-wide | 11573(47.83%) | 12623(52.17%) |
| TF binding | 5055(51.87%) | 4691(48.13%) |

^a^Positive ∆*∆G_s_* indicated the increased base pair stacking interactions after mutation. ^b^Negative ∆*∆G_s_* indicated the decreased base pair stacking interactions after mutation.

Table S5**.** Binding free energy values of the different systems estimated by the MM/GBSA calculations (kcal/mol).

| Type | Mutation Site | Binding Affinity | ∆*H_b_* | Entropy  (310K) | ∆*G_b_* | ∆∆*G_b_* |
| --- | --- | --- | --- | --- | --- | --- |
| Wild-type | A | / | -134.88 ±0.18 | -99.78 ±3.36 | -35.10 | / |
| Mutated-type | G | Increased | -141.15 ±0.17 | -104.63 ±2.94 | -36.52 | -1.42 |

The table shows the binding free energies of the wild-type and mutated complexes. The binding affinity of the wild-type and mutated complexes were obtained from experiment (Clamp et al., 2007) The binding free energy of the MEIS1 complexes were calculated by MM/GBSA (Miller et al., 2012). The entropy of the MEIS1 complexes was calculated using Normal Mode Analysis (Nmode) in AmberTools

**References:**

Clamp, M., Fry, B., Kamal, M., Xie, X., Cuff, J., Lin, M. F., Kellis, M., Lindblad-Toh, K., and Lander, E. S. (2007). Distinguishing protein-coding and noncoding genes in the human genome. *Proc Natl Acad Sci U S A* **104**, 19428-19433. doi: 10.1073/pnas.0709013104

Miller, B. R., 3rd, McGee, T. D., Jr., Swails, J. M., Homeyer, N., Gohlke, H., and Roitberg, A. E. (2012). MMPBSA.py: An Efficient Program for End-State Free Energy Calculations. *Journal of Chemical Theory and Computation* **8**, 3314-3321. doi: 10.1021/ct300418h
